# Fatigue and Muscle Atrophy in a Mouse Model of Myasthenia Gravis Is Paralleled by Loss of Sarcolemmal nNOS

**DOI:** 10.1371/journal.pone.0044148

**Published:** 2012-08-28

**Authors:** Sarina Meinen, Shuo Lin, Markus A. Rüegg, Anna Rostedt Punga

**Affiliations:** Department of Neurobiology/Pharmacology, Biozentrum, University of Basel, Basel, Switzerland; Albany Medical College, United States of America

## Abstract

Myasthenia Gravis (MG) patients suffer from chronic fatigue of skeletal muscles, even after initiation of proper immunosuppressive medication. Since the localization of neuronal nitric oxide synthase (nNOS) at the muscle membrane is important for sustained muscle contraction, we here study the localization of nNOS in muscles from mice with acetylcholine receptor antibody seropositive (AChR+) experimental autoimmune MG (EAMG). EAMG was induced in 8 week-old male mice by immunization with AChRs purified from *torpedo californica*. Sham-injected wild type mice and *mdx* mice, a model for Duchenne muscular dystrophy, were used for comparison. At EAMG disease grade 3 (severe myasthenic weakness), the triceps, sternomastoid and masseter muscles were collected for analysis. Unlike in *mdx* muscles, total nNOS expression as well as the presence of its binding partner syntrophin α-1, were not altered in EAMG. Immunohistological and biochemical analysis showed that nNOS was lost from the muscle membrane and accumulated in the cytosol, which is likely the consequence of blocked neuromuscular transmission. Atrophy of all examined EAMG muscles were supported by up-regulated transcript levels of the atrogenes atrogin-1 and MuRF1, as well as MuRF1 protein, in combination with reduced muscle fiber diameters. We propose that loss of sarcolemmal nNOS provides an additional mechanism for the chronic muscle fatigue and secondary muscle atrophy in EAMG and MG.

## Introduction

Myasthenia Gravis (MG) is an autoimmune disorder where autoantibodies target the nicotinic acetylcholine receptors (AChR) at the neuromuscular junction (NMJ) in about 85% of patients [1]. These antibodies cause impaired neuromuscular transmission, resulting in the cardinal symptoms of fluctuating skeletal muscle weakness of predominantly proximal muscles in the face, neck, arms and legs. Treatment consists of immunosuppressive medication along with symptomatic treatment, including acetylcholinesterase inhibitors (AChEI), which renders the neurotransmitter ACh available for longer time at the NMJ and thus temporarily improves the neuromuscular transmission. Due to the beneficial effects of the β2 adrenergic receptor (β2AR) agonist terbutaline on muscle fatigue in MG patients, this drug has been used as symptomatic treatment in a few neurology clinics [2], [3](Punga AR, unpublished observations). β2ARs are G protein coupled receptors, and stimulation by β2AR agonists such as salbutamol increases inctracellular levels of cyclic AMP and activates the cyclic guanosine monophosphate (cGMP) pathway [4]–[6].

Nitric oxide (NO) is a signaling molecule involved in vital physiological processes, such as neurotransmission and gene regulation, by increasing intracellular levels of cGMP. In turn, cGMP is inactivated by phosphodiesterases (PDEs), multi-domain proteins with distinct catalytic and regulatory sites. The rat model of EAMG is characterized by an increase of PDE subtypes in both lymph nodes and in muscles [7]. Pentoxifylline, a general PDE inhibitor, inhibits the progression of rat EAMG, suggesting the involvement of PDE regulation in EAMG pathogenesis [7]. Additional studies have shown the up-regulation of PDE also in human MG, but also in other autoimmune disorders such as multiple sclerosis [8]. NO synthase (NOS) catalyzes the production of NO and is present in three different isoforms: 1) neuronal NOS (nNOS), expressed in for example motor neurons, skeletal and smooth muscles 2) inducible NOS (iNOS), expressed in most cells after immunological or inflammatory stimuli and 3) endothelial NOS (eNOS), expressed in the endothelium. The neuronal form nNOS is also expressed in fast-twitch fibers of skeletal muscles and localizes to the cytosolic surface of the sarcolemma, where it binds to syntrophin α-1, a component of the dystrophin-glycoprotein complex. Upon muscle contraction, nNOS is stimulated to induce vasodilatation through regulation of the local blood flow in the muscle and thus increases blood supply of active muscles [9]. The localization of nNOS at the sarcolemma is essential for instant diffusion of NO to muscle vasculature where it induces vessel dilatation via the cGMP pathway [10], [11]. Denervation has been reported to cause dissociation of nNOS from the sarcolemma, resulting in muscle fatigue due to absence of nNOS-cGMP signaling [12]–[15]. Moreover, dissociation of nNOS from the sarcolemma increases the NO availability in the cytosol, which in turn causes up-regulation of the atrophy-inducing atrogenes MuRF1 and atrogin-1 [15]. In the *mdx* mice, representing a model of Duchenne muscular dystrophy, nNOS and its binding partner syntrophin α-1 are absent from the sarcolemma due to failure of assembly of the entire dystrophin-glycoprotein-complex [16].

One of the puzzling questions is why the majority of MG patients continue to have chronic fatigue despite proper immunosuppressive medication that should remove the circulating autoantibodies and inhibit the T-and B-cell response. MG is generally regarded as a disorder with no pathologic alterations of the muscle fiber metabolism, although muscle atrophy, especially of type II fibers, is known to arise in a large proportion of MG patients [17]–[19]. Hence, additional mechanisms are suspected to play a role.

In this work, we investigated the possibility of an alternative pathway/mechanism, other than blocked neuromuscular transmission, to explain the occurrence of post-exercise fatigue in skeletal muscles in many MG patients on proper immunosuppressive therapy. We show that nNOS was lost from the muscle membrane and accumulated in the cytosol of muscle fibers from mice with AChR+ EAMG. Notably, the atrophy-related atrogenes MuRF1 and atrogin-1 as well as the denervation marker AChRγ were highly up-regulated in all the muscles examined, which has not previously been reported in EAMG with AChR antibodies. Our data thus provide evidence for an additional mechanism that might be involved in muscle fatigue and atrophy observed in EAMG mice and in a large fraction of MG patients.

## Methods

### Experimental Animals

Wild type C57BL6 mice and dystrophic *mdx* mice [20] were originally supplied from Jackson Laboratories (Bar Harbor, Maine, US). For immunization, 8 week-old male C57BL6 mice and 3 month-old male dystrophic *mdx* mice were used. All mice were housed in the Animal Facility of Biozentrum, University of Basel, where they had free access to food and water in a room with controlled temperature and a 12-hour alternating light–dark cycle. All animal procedures complied with Swiss animal experimental regulations (ethical application approval no. 2352) and EC Directive 86/609/EEC.

**Figure 1:**
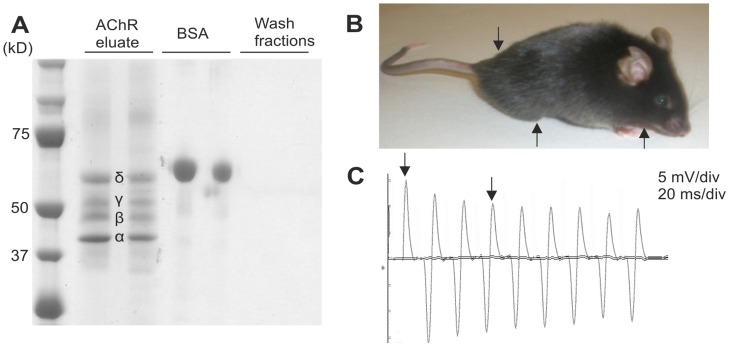
Immunization of mice with purified AChRs causes production of autoantibodies along with clinical and neurophysiological signs of experimental autoimmune MG (EAMG). (A) The purification of AChRs from the electric organs of *torpedo californica* contained the subunits of: δ, γ, β and α at their corresponding sizes. (B) The typical clinical presentation in AChR+ mice with EAMG disease grade 3 was flaccid paralysis, with inability to rise up on the hind limbs, tail down and chin down (arrows). (C) The mice were examined with low frequency (3 Hz) repetitive nerve stimulation of the sciatic nerve. A representative decrement, “run down” (arrows), of the compound motor action potentials (CMAPs) of ∼30% was noted between the 1^st^ and 4^th^ CMAP in the EAMG mice. A decrement exceeding 10% was considered pathological.

**Figure 2:**
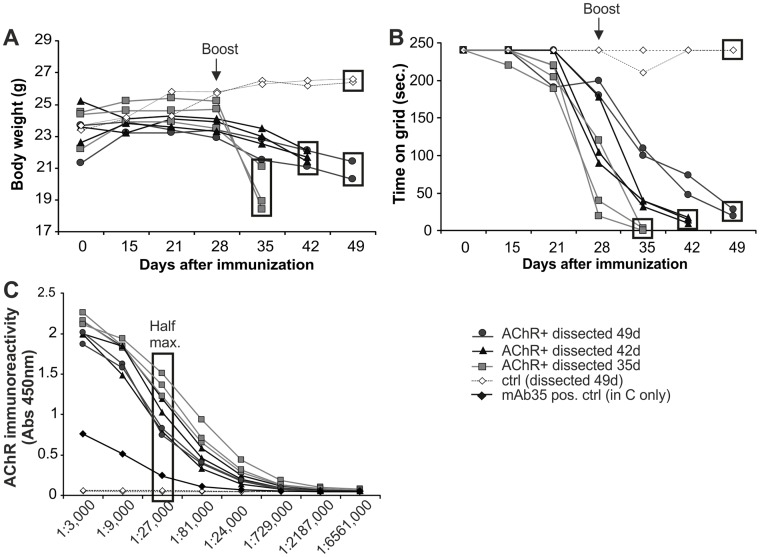
Clinical characteristics of EAMG mice that developed a severe disease phenotype (EAMG grade 3; n = 8). (A) Loss of body weight in the AChR+ EAMG mice started after day 28. Day of dissection is indicated by a square (35, 42 or 49 days after post-immunization). (B) The time AChR+ EAMG mice could hold on to the upside-down grid is displayed. Mice with a grid time <0 seconds (EAMG grade 3) were sacrificed for analysis. The day of dissection is indicated by a square. (C) ELISA analyzed the immunoreactivity of AChR autoantibodies in sera from immunized mice. The background from negative controls was subtracted and anti-AChR antibody mAb35 was used as positive control. Half maximum titer is indicated. Control mice (ctrl) were immunized with CFA/PBS.

### Immunization of Mice with AChR from Torpedo Californica

AChRs were purified from the electric organs of *torpedo californica* using cobratoxin affinity columns as described previously [21] and were tested for integrity by SDS-PAGE. 18 C57BL6 male mice aged 8 weeks were anesthetized (Ketamine: 111 mg/kg and Xylazine: 22 mg/kg) and immunized with 20 µg of AChR emulsified in complete Freund’s adjuvant (CFA, Difco laboratories) subcutaneously in the hind foot pads, at the base of the tail and dorsolateral on the back. At day 28 post-injection, immunization was repeated. Control mice (5 male mice) were immunized with PBS/CFA at day 0 and day 28 (similar to the EAMG immunization protocol).

**Figure 3:**
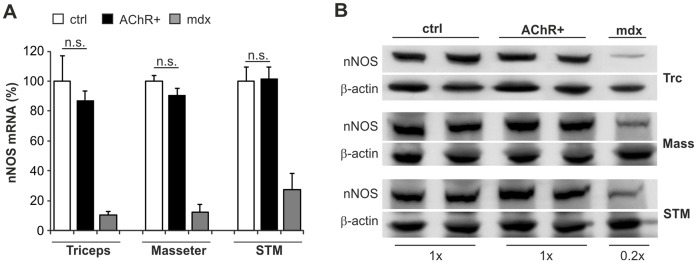
Total transcript and protein levels of nNOS are normal in muscles of EAMG mice. (A) Total mRNA amount of nNOS in triceps, masseter and sternomastoid muscles. Negative controls were muscles from healthy CFA/PBS injected control mice (ctrl; n = 3) and as positive control muscles from *mdx* mice were used (*mdx*; n = 3). In the AChR+ EAMG muscles (AChR+; n = 6), total nNOS levels were unchanged as compared to controls (triceps p = 0.55; triceps p = 0.32; triceps p = 0.90). nNOS mRNA was clearly reduced in *mdx* muscles. (B) Western blot analysis of nNOS (160 kD) levels in total protein extracts from triceps (Trc), masseter (Mass) and sternomastoid (STM) muscles. Representative results are shown. No difference was seen between nNOS protein content in AChR+ EAMG (AChR+; n = 3) and control (ctrl; n = 3) muscles, whereas nNOS protein was reduced in muscles of *mdx* (*mdx*; n = 3)-mice to approximately 20%. β-actin was used as reference protein.

### Clinical Examination

Body weight was monitored weekly. Mice were provided with soft, wet food in the cage to minimize the contribution of insufficient food and water intake to the body weight loss. Muscle fatigue/weakness of the immunized mice was assessed once a week, as previously described [22]. Briefly, mice were exercised by 20 consecutive front and hind paw grips on a vertical grid, gently held at the base of their tail. The mice were then placed on an upside-down grid. The time they could hold on to the grid reflected the grade of fatigue and muscle weakness. EAMG grades were as follows: Grade 0, no fatigue (>150 sec), normal posture; Grade 1, mild muscle fatigue after exercise (<60 sec), normal posture; Grade 2, moderate muscle fatigue (<30 sec), change to hunched posture with body weight loss and decreased activity; Grade 3, severe generalized weakness (<0 sec), generalized weakness, hunched posture with flaccid paralysis, obvious loss of body weight, moribund. The following muscles were obtained from mice with EAMG grade 3 (n = 8): triceps, masseter and sternomastoid muscles. Repetitive nerve stimulation of the sciatic nerve was done as previously described [23].

**Figure 4:**
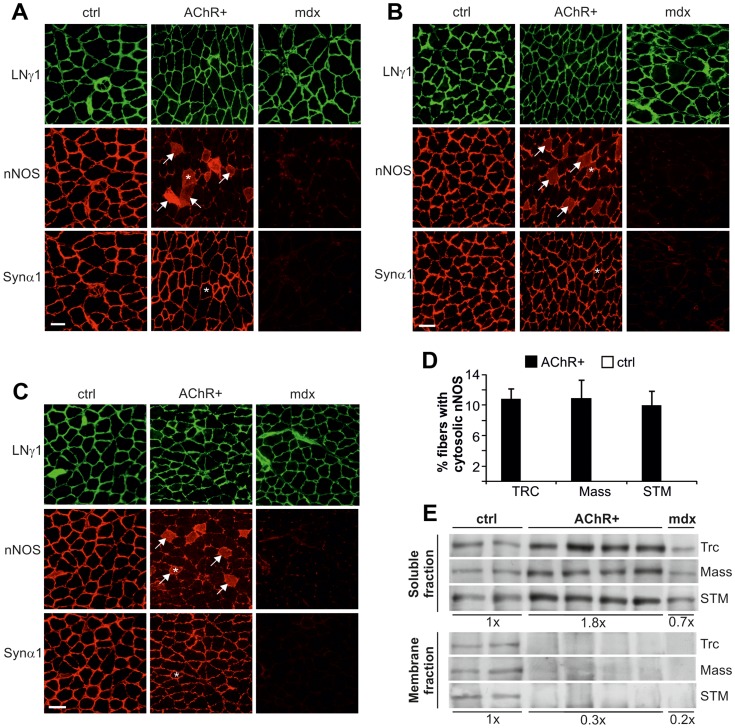
nNOS is lost from the sarcolemma and accumulates in the cytosol in muscles of EAMG mice. Immunostaining of cross-sections from (A) triceps, (B) masseter and (C) sternomastoid muscles with laminin-γ1 (LNγ1; first panels), nNOS (second panels) and syntrophin-α1 (Synα1; third panels) antibodies. Representative images are shown. In contrast to CFA/PBS injected control mice (ctrl; n = 3 for each muscle), sarcolemmal nNOS staining partially disappeared from the sarcolemma and was instead present in the cytosol (arrows) in AChR+ EAMG muscles (AChR+; n = 4 for each muscle). Syntrophin α-1, the binding partner of nNOS, stably remained at the membrane. nNOS and syntrophin α-1 both were almost absent in *mdx* muscles (n = 2 for each muscle). Asterisks indicate identical fibers in consecutive sections. (D) 10–11% of the fibers in AChR+ EAMG muscles were positive for cytosolic nNOS staining, whereas no such fiber was found in control muscles. (E) Western blot analysis of the nNOS protein levels present in the soluble (cytosol; upper panel) and the membrane (sarcolemma; lower panel) protein fraction of triceps (Trc), masseter (Mass) and sternomastoid (STM) muscles. Representative bands are shown. nNOS levels were increased in the soluble fraction of muscles from AChR+ EAMG mice (AChR+; n = 6) with approximately 80% when compared to muscles from CFA/PBS injected control mice (ctrl; n = 4). In *mdx* mice (n = 3), approximately 70% of nNOS protein remained in the cytosol. In the membrane fraction, indicating sarcolemmal position, nNOS was reduced to 30% of control values in AChR+ EAMG mouse muscles and to 20% in *mdx* muscles. Scale bars  = 50 µm.

### ELISA

Sera were obtained from tail vein blood at the day of dissection (day 35 to 49 post-immunization). ELISA plates (Nunc MaxiSorp, Fisher Thermo Scientific) were coated with 50 µl/well of recombinant purified AChRs (8 ng/ml), blocked with 3% BSA/PBS and then incubated with a sera dilution row (1∶3000–1∶6561,000). After washing, plates were incubated with secondary HRP-conjugated goat-anti-mouse (1∶2000; Jackson Immuno Research Laboratories). HRP was activated by a TMB substrate and the reaction was stopped with 1N HCl. Absorbance was read at 450 nm. Sera from CFA/PBS injected mice constituted negative controls and monoclonal mouse anti-AChR α subunit antibody; mAb35 [24] served as positive control.

**Figure 5:**
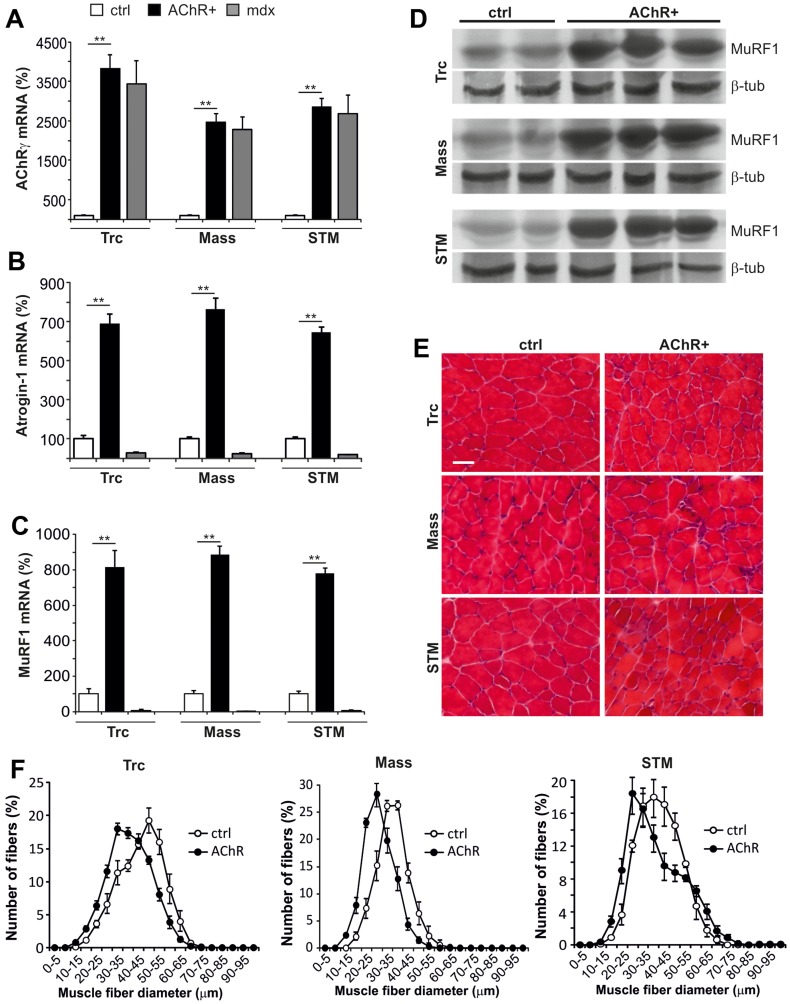
Denervation and atrophy in triceps (Trc), masseter (Mass) and sternomastoid (STM) muscles of EAMG mice. (A) mRNA levels of AChRγ subunit, a marker for blocked neuromuscular transmission and denervation, were up-regulated 25–40 times (p<0.005 for all muscles) in muscles of AChR+ EAMG mice (n = 6) when compared to controls (ctrl; n = 3). (B) mRNA levels of the atrophy-related gene atrogin-1 were 6.5–7.5 times increased in AChR+ EAMG (n = 6) compared to control (ctrl; n = 3; p<0.005 for all muscles). (C) mRNA levels of the atrophy inducing gene MuRF1 were around 8 times higher in AChR+ EAMG (n = 6) compared to control (ctrl; n = 3; p<0.005 for all muscles). In *mdx* muscles (n = 3) atrogin-1 and MuRF1 mRNA was down-regulated compared to control mice. (D) Western blot analysis shows increased MuRF1 protein levels in AChR+ EAMG muscles. β-tubulin (β-tub) was used as loading control. (E) H&E staining visualizes smaller atrophied fibers in AChR+ EAMG than in control muscles. (F) Muscle fiber size distribution shifts for 5 to 15 µm to smaller fibers in AChR+ EAMG muscles, indicating atrophy (N = 4). Values represent relative numbers of fibers in a given diameter class (5 µm/class).

### Quantitative PCR Analysis

Mouse muscle RNA was extracted and purified as previously described [23]. Equal amounts of total RNA were used for cDNA synthesis (iScript cDNA Synthesis Kit, BioRad). RT-PCR reactions (triplicates) were performed using Power SYBR Green PCR Master Mix reagent (Applied Biosystems, Warrington, UK). β-actin served as endogenous control. The following primer sets were used:

nNOS: 5′-GGG CAA ACA GTC TCC TAC CA-3′ and 5′-AGG GTG TCA GTG AGG ACC AC-3′; atrogin-1∶5′-CTC TGT ACC ATG CCG TTC CT-3′ and 5′-GGC TGC TGA ACA GAT TCT CC-3′; MuRF1∶5′-ACC TGC TGG TGG AAA ACA-3′ and 5′-AGG AGC AAG TAG GCA CCT CA-3′; AChR-γ subunit: 5′-AAC GAG ACT CGG ATG TGG TC-3′ and 5′-GTC GCA CCA CTG CAT CTC TA-3′; β-actin: ′-CAG CTT CTT TGC AGC TCC TT-3′ and 5′-GCA GCG ATA TCG TCA TCC A-3′.

**Figure 6:**
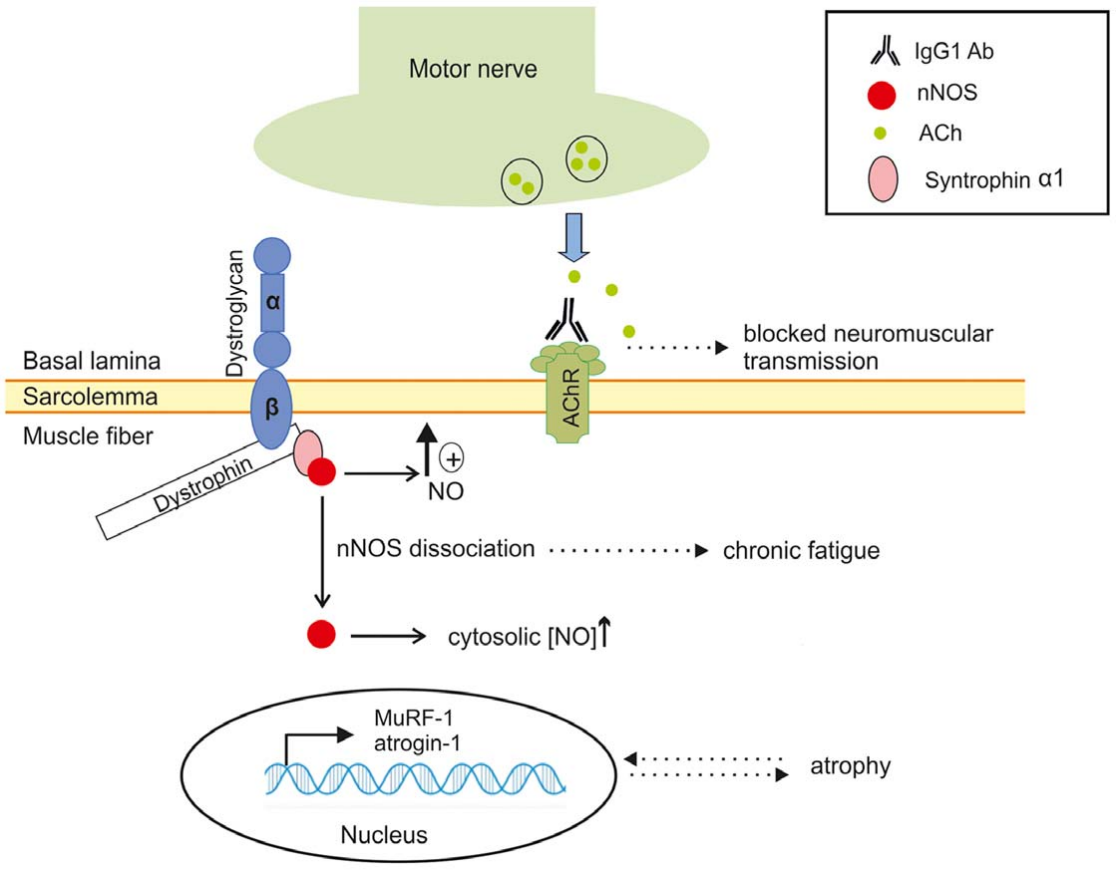
Proposed model of translocation of sarcolemmal nNOS to the cytosol and its consequences in EAMG. In normal muscle fibers, where nNOS is present at the sarcolemma, localized NO production at the membrane is essential for sustained muscle contraction through activation of cyclic GMP. Antibodies (IgG1) against the nicotinic acetylcholine receptors (AChRs) at the muscle membrane cause impaired neuromuscular transmission and functional denervation of some muscle fibers. The consequential dissociation of nNOS from its binding partner syntrophin-α1 to the cytosol causes an up-regulation of atrogene transcription (MuRF1 and atrogin-1). Lack of sarcolemmal nNOS signaling is believed to contribute to muscle fatigue by impeding cGMP-mediated vasodilatation, while the atrogene up-regulation in turn is believed to cause secondary muscle atrophy.

### Total Protein Extraction and Protein Fractionation

Triceps, masseter and sternomastoid muscles were pulverized on a liquid nitrogen-cooled metal plate and total protein was extracted by homogenization in RIPA buffer containing protease inhibitors (Roche Diagnostics). The subcellular fractionation was done as previously described [16]. In brief, pulverized muscles were homogenized in 10 volumes (w/v) of buffer A (25 mM Tris–HCl, pH 7.4, 100 mM NaCl, 1 mM EDTA, 1 mM EGTA) containing protease inhibitors (Roche Diagnostics) followed by centrifugation at 1000×g to pellet the nuclei. The supernatant was then centrifuged at 20,000×g to yield the supernatant S1, referring to the soluble fraction. The pellet from this centrifugation was resuspended in buffer B (500 mM NaCl added to buffer A), incubated for 30 min at 4°C with agitation and then centrifuged at 15,000×g for 30 min, yielding supernatant S2 (microsomal membrane fraction). The pellet from this last centrifugation was further resuspended in buffer B, containing 0.5% Triton X-100, incubated for 30 min at 4°C with agitation, and centrifuged at 15,000×g for 30 min to get supernatant S3 (cytoskeletal fraction) and the final pellet, P (sarcolemma). The fractions were resolved using the loading buffer (Laemmli) and analyzed by SDS-gel electrophoresis.

### Western Blot Analysis

Protein concentration of the total protein fraction, the soluble fraction S1 and the sarcolemmal final pellet P was determined using BCA Protein assay kit (PIERCE). Western blot of triceps, masseter and sternomastoid muscles was conducted as described [25]. 20 µg of total protein was resolved on a 4–12% Nu-PAGE Bis-Tris gel (Invitrogen) and then transferred to nitrocellulose membrane. Similarly, 20 µg of protein fraction from each muscle was separated on a 6% Laemmli SDS-polyacrylamid gel and then transferred to a nitrocellulose membrane. The membranes were probed with rabbit polyclonal anti-nNOS (160 kD, 1∶500, Invitrogen 61–7000) and rabbit monoclonal anti-β-actin (45 kD, 1∶1000, NEB) or with rabbit polyclonal anti-MuRF1/TRIM63 MaxPab (40 kD, 1∶1000, Abnova D01) and rabbit polyclonal anti-β-tubulin (55 kD, 1∶500, Abcam ab6046). Bound antibodies were then recognized with HRP-conjugated antibody (1∶5000; ICL). Chemiluminescence was performed using LumiGLO Chemiluminescent Substrate System (KPL, 54-61-00). Quantification of band densitometry was done in ImageJ, with normalization to the reference β-actin or β-tubulin.

### Immunostainings and H&E Staining

Muscles were snap-frozen in liquid nitrogen-cooled isopentane (–150°C), embedded in 7% gum Tragacanth (Sigma) and cross-sections of 12-µm thicknesses were cut. General histology was performed using Hematoxylin & Eosin staining (H? Merck). The antibodies used for immunofluorescence were purchased from the following commercial sources: rabbit anti-nNOS (1∶100, Invitrogen), rabbit anti-syntrophin α-1 (1∶500, GeneTex GTX11187) and rat anti-laminin-γ1 B2 chain (1∶500, Millipore MAB1914). Membrane-bound and extracellular epitopes were visualized with Alexa-488-conjugated wheat germ agglutinin (WGA; Molecular Probes, Eugene, OR, USA). Secondary antibodies were Cy3-conjugated donkey anti-rabbit (BioLegend, 406402) and Dy-Light 488 goat anti-rat (BioLegend, 405409). Pictures of stained, consecutive cross-sections were collected using a Leica DM5000B fluorescence microscope, a digital camera (F-View; Soft Imaging System), and analySIS® software (Soft Imaging System).

### Histological Quantifications

The percentage of fibers with cytosolic nNOS staining in AChR+ EAMG muscles was evaluated by counting a total of 300 muscle fibers per section. The muscle fiber size distribution was quantified on WGA-stained cross-sections using the minimum distance of parallel tangents at opposing particle borders (minimal “Feret’s diameter”) as described [26]. Normalization of the number of fibers in each fiber Feret class of 5 µm was based on the total fiber number measured per muscle.

### Statistical Analysis

Quantitative data are expressed as mean ± SEM. *P*-values were calculated using the unpaired two-sample Student *t*-test assuming equal variances and the significance level was defined as p<0.05.

## Results

### Fatigue and Weight Loss in AChR+ EAMG

AChRs were affinity-purified from the electric organ of *torpedo californica* using cobratoxin-coupled Sepharose beads [21]. To test for purity, eluates were analyzed by SDS-PAGE followed by Coomassie-blue staining. All four AChR subunits (α,β,δ,γ) were present at their expected sizes, while the last wash fractions of the columns were free of any protein (Fig. 1A). Eighteen mice were immunized with the purified AChRs in CFA. Out of these mice, 1 mouse (5%) unexpectedly died during the course of the experiment, 5 mice (28%) did not develop any myasthenic symptoms (grade 0), 3 mice (17%) developed mild fatigue upon exercise (grade 1), 1 mouse (5%) reached EAMG disease grade 2 and the remaining 8 mice (45%) developed severe EAMG (grade 3) at various time points from 35 days up to 49 days. The phenotype of EAMG grade 3 included severe myasthenic weakness with hunched posture, flaccid paralysis and obvious weight loss [27] (Fig. 1B). Impaired neuromuscular transmission was confirmed by decrement of the compound motor action potentials (CMAP) on *in vivo* repetitive sciatic nerve stimulation (Fig. 1C). Disease severity progressed after the 2^nd^ boost at day 28, with weight loss (Fig. 2A) and muscle fatigue upon exercise on an upside-down grid [22] (Fig. 2B). Both parameters correlated well with the disease grade and could therefore be used to follow disease progression in the EAMG mice. None of the control mice (n = 5) displayed any weight loss or muscle fatigue (>150 s on the grid). Immunized mice that developed obvious severe myasthenic symptoms (n = 8) were sacrificed one to three weeks after the boost (day 35 to day 49 post-immunization), when reaching EAMG grade 3. In these mice, an elevated titer of AChR antibodies was detected in the sera, which were collected at the day of euthanization. AChR antibodies were present in sera from all examined EAMG mice with severe myasthenic weakness and the titers were higher than the titer measured from the positive control (anti-AChR antibody mAb35; Fig. 2C).

### Normal Transcript and Protein Levels of nNOS in Muscles of EAMG Mice

Since triceps, masseter and sternomastoid muscles represent proximal and facial muscles that are often affected by myasthenic fatigue, these muscles were examined for their total mRNA and protein levels of nNOS. Total nNOS transcript levels in muscles of AChR+ EAMG mice with disease grade 3 were comparable to those from muscles of healthy control mice injected with CFA/PBS (ctrl; Fig. 3A). In contrast and as previously reported [28], muscles from *mdx* mice, a model of Duchenne muscular dystrophy, showed a strong reduction of nNOS mRNA expression (Fig. 3A). Similar results were obtained by Western blot analysis in total muscle protein extracts from the triceps, masseter and sternomastoid muscles. There, total protein levels of nNOS in AChR+ EAMG muscles were the same as in control muscles, but again nNOS was significantly reduced in muscles from *mdx* mice (Fig. 3B). Thus, the total amount of nNOS in AChR+ EAMG muscles is comparable to control muscles, in contrast to the strongly reduced nNOS levels in muscles from *mdx* mice.

### nNOS is Lost from the Sarcolemma and Accumulates in the Cytosol in EAMG

To examine the subcellular localization of nNOS in AChR+ EAMG mice, cross-sections of triceps (Fig. 4A), masseter (Fig. 4B) and sternomastoid (Fig. 4C) muscles were stained for nNOS (second row in each figure) and for the basement membrane protein laminin-γ1 (LN1 γ; first panel). Whereas the sarcolemmal staining for nNOS was strong in control mice, it was partially or completely absent in some fibers of the muscles in AChR+ EAMG mice (center panel in each of Fig. 4A, B, C). In addition, several muscle fibers showed an increase in cytosolic staining for nNOS (Fig. 4A, B, C), indicating loss of nNOS from the sarcolemma to the cytosol in those fibers. In agreement with previous reports [16], the amount of nNOS was strongly reduced in all examined muscles from the *mdx* mice. To test whether nNOS was lost from the sarcolemma in AChR+ EAMG mice because of displacement of its binding partner syntrophin α-1, consecutive sections were stained for syntrophin α1 (third row of panels in Fig. 4A, B, C). In contrast to *mdx* muscles, where syntrophin α-1-immunoreactivity was absent at the sarcolemma, syntrophin-α1 was still detected at the sarcolemma of AChR+ EAMG muscles and its levels were comparable to those from control muscles.

To obtain an estimate of the extent of nNOS that was lost from the sarcolemma into the cytosol, the percentage of fibers with cytosolic nNOS staining was counted in AChR+ EAMG muscles (Fig. 4D). A clear cytosolic signal for nNOS could be detected in 10–11% of EAMG muscle fibers, whereas no fibers positive for nNOS in the cytosol were found in control muscles or in muscles from *mdx* mice.

To test whether nNOS that disappeared from the sarcolemma could still be found in the cytosol, we next performed subcellular fractionation of the muscle followed by Western blot analysis. While the amount of nNOS was approximately the same in the soluble (cytosol) and the membrane (sarcolemma) fraction in control mice, the majority of nNOS was detected in the soluble fraction in both AChR+ EAMG and *mdx* mice (Fig. 4E). These results indicate that the connection of nNOS to the sarcolemma becomes very fragile in AChR+ EAMG muscles and thus nNOS is lost from the membrane and accumulates in the cytosol.

### Functional Denervation and Muscle Atrophy Parallels the Loss of Sarcolemmal nNOS in EAMG

Dissociation of sarcolemmal nNOS has previously been reported in mice upon hind-limb suspension and denervation [15]. To examine whether partial denervation may also induce nNOS dissociation from the sarcolemma in AChR+ EAMG, mRNA levels of the fetal AChRγ subunit, a marker for blocked neuromuscular transmission and denervation, were determined. Indeed, AChRγ was significantly up-regulated in AChR+ EAMG muscles (Fig. 5A), suggesting that loss of sarcolemmal nNOS arises from functional denervation, which could be mediated by the AChR blocking autoantibodies. The up-regulation of AChRγ in muscle fibers of *mdx* mice was in support of previous studies of the diaphragm and interosseus muscles [29], [30].

Further, nNOS accumulation in the cytosol has been linked to promotion of muscle atrophy by up-regulation of the E3 ligases [15]. Indeed, mRNA levels of the two atrophy-associated genes atrogin-1 (Fig. 5B) and MuRF1 (Fig. 5C) were highly up-regulated in all examined EAMG muscles. Accordingly, MuRF1 protein levels were also increased (Fig. 5D). In contrast, mRNA levels of both atrogenes were rather reduced in *mdx* muscles (Fig. 5B–C), which can be explained by the so-called “pseudohypertrophy” that develops in the *mdx* mice as well as in patients with Duchenne muscular dystrophy. Muscle fiber atrophy was visualized in H&E stained EAMG muscle cross-sections (Fig. 5E) and a quantitative evaluation of the fiber size distribution confirmed the shift to smaller fibers in AChR+ EAMG compared to control muscles (Fig. 5F). In summary, denervation marker and atrophy factors were up regulated and muscle fiber atrophy was evident in all examined muscles of EAMG mice.

## Discussion

The clinical hallmark of AChR+ MG patients, as well as AChR+ EAMG mice, is a varying degree of fatigable weakness of proximal skeletal muscles. Since many MG patients continue to have post-exercise fatigue of skeletal muscles although proper immunosuppressive treatment has been initiated, the question arises of whether additional mechanisms contribute to atrophy or blocked neuromuscular transmission.

Loss of nNOS from the sarcolemma is found in muscles from patients with distinct myopathies and there is an association of whole-body fatigue with loss of sarcolemmal nNOS in mice [12]. Although MG is generally regarded as a disorder with no pathological alterations of the muscle fiber metabolism or structure, the present findings of nNOS translocation from the membrane to the cytosol in AChR+ EAMG mice, suggest a common mechanism of fatigue in myasthenia and myopathies. In fact, this mechanism is likely to contribute to the muscle fatigue response to exercise, since sarcolemma-localized nNOS signaling in skeletal muscle is required to maintain cGMP-mediated vasodilation of contracting muscles [11].

β2AR agonists, mainly terbutaline, have been reported to ameliorate the clinical symptoms in MG patients [3] and recently this group of medication (albuterol) was recognized to improve clinical symptoms also in patients with congenital myasthenic syndromes (CMS) [31]. A randomized placebo-controlled pilot study of terbutaline in MG patients showed a significant improvement in clinical quantitative fatigue score as well as an improvement in the fatigue response on repetitive nerve stimulation [2]. Stimulation with salbutamol is known to result in an accumulation of cGMP in the arterial mesenteric bed, indicating that activation of β2-ARs is coupled to the stimulation of cGMP production by the arterial mesenteric bed along with increased NO release [4], [5]. Hence, β2-AR agonist-induced vasodilatation could provide a basis for improvement of fatigue in MG patients, through increasing blood flow to the skeletal muscles by enhancing NO-cGMP signaling. In the rat model of EAMG, several PDE subtypes were up regulated both in the lymph nodes and in the muscles and the general PDE inhibitor pentoxifylline inhibited the progression of EAMG [7], [8]. Hence, since inhibition of PDE increases the amount of intracellular cGMP this could explain why less muscle involvement is found in EAMG rats upon this treatment [7].

After denervation of adult muscles the embryonic-type AChRs, AChRγ, are again expressed over the entire fiber length and disappear upon reinnervation [32]. Our data indicate a denervation process in AChR+ EAMG mice. Up-regulation of the embryonic AChRs has previously been reported in fibers from both the interosseus and diaphragm muscles in *mdx* mice, similar to our findings, indicating a denervation/regeneration process of the dystrophin-deficient muscle fibers [29], [30]. Since dissociation of nNOS from the sarcolemma can be induced by denervation or “muscle unloading” [15], the up-regulation of the denervation marker AChRγ indicates that the weak connection of nNOS to the sarcolemma in AChR+ EAMG mice may arise from impaired neuromuscular transmission caused by the antibody-mediated blockade of AChRs. Further, we provide evidence that in contrast to *mdx* muscles, in which both nNOS and its binding partner syntrophin-α1 are absent from the sarcolemma due to failure of assembly of the entire dystrophin-glycoprotein-complex, syntrophin-α1 deficiency is not the reason for nNOS loss at the sarcolemma in EAMG mice.

Mice deficient for nNOS appear normal regarding activity, breeding and memory [33] and no muscle pathology nor loss of muscle force has been observed [12]. Nevertheless, nNOS knockout mice display a deficit in their adaptation to exercise and thus experience muscle fatigue [14]. However, the low levels of nNOS in *mdx* mice both at the mRNA and the protein level in whole muscle extracts were in contrast to the AChR+ EAMG mice, where the reduced sarcolemmal nNOS was seen in parallel with a significantly increased cytosolic nNOS level. As reported in other neuromuscular diseases such as amyotrophic lateral sclerosis [34], an excess of NO production in the cytosol boosts muscle atrophy through action on the atrogenes Foxo3a, MuRF1 and atrogin-1 [15]. The significant up-regulation of these atrophy markers have previously been described only in facial muscles of EAMG mice with antibodies against muscle specific tyrosine kinase (MuSK), however our data indicate that muscle atrophy occurs at an early stage also in AChR+ EAMG. This in turn most likely causes a “vicious circle”, where the presence of cytosolic nNOS causes activation of the E3 ligases [15], resulting in aggravated atrophy and chronic fatigue (Fig. 6). The AChR+ EAMG model is naturally more dramatic than the typical gradual onset of MG in human patients, which could in part explain the strong up-regulation of the atrogenes MuRF1 and atrogin-1 as well as the denervation marker AChRγ. Notwithstanding, type II fiber atrophy has been recognized in a large proportion of MG patients [19] and this is often referred to as “disuse atrophy”.

In summary, we show that nNOS is lost from the sarcolemma and accumulates in the cytosol in muscle fibers of EAMG mice. Absence of sarcolemmal nNOS represents a possible mechanism for the chronic fatigue experienced by AChR+ MG patients. In addition, increased nNOS availability in the cytosol provides a potential explanation for the muscle atrophy in patients with long-term severe MG weakness.
